# An Epistatic Network Describes *oppA* and *glgB* as Relevant Genes for *Mycobacterium tuberculosis*

**DOI:** 10.3389/fmolb.2022.856212

**Published:** 2022-05-31

**Authors:** Ali-Berenice Posada-Reyes, Yalbi I. Balderas-Martínez, Santiago Ávila-Ríos, Pablo Vinuesa, Salvador Fonseca-Coronado

**Affiliations:** ^1^ Posgrado en Ciencias Biológicas, UNAM, Mexico, Mexico; ^2^ Facultad de Estudios Superiores Cuautitlán, UNAM, Estado de Mexico, Mexico; ^3^ Instituto Nacional de Enfermedades Respiratorias “Ismael Cosio Villegas”, Ciudad de Mexico, Mexico; ^4^ Centro de Ciencias Genómicas, UNAM, Cuernavaca, Mexico

**Keywords:** *glgB*, *oppA*, epistatic network, co-selection, *Mycobacterium tuberculosis*, tuberculosis

## Abstract

*Mycobacterium tuberculosis* is an acid-fast bacterium that causes tuberculosis worldwide. The role of epistatic interactions among different loci of the *M. tuberculosis* genome under selective pressure may be crucial for understanding the disease and the molecular basis of antibiotic resistance acquisition. Here, we analyzed polymorphic loci interactions by applying a model-free method for epistasis detection, SpydrPick, on a pan–genome-wide alignment created from a set of 254 complete reference genomes. By means of the analysis of an epistatic network created with the detected epistatic interactions, we found that *glgB* (*α*-1,4-glucan branching enzyme) and *oppA* (oligopeptide-binding protein) are putative targets of co-selection in *M. tuberculosis* as they were associated in the network with *M. tuberculosis* genes related to virulence, pathogenesis, transport system modulators of the immune response, and antibiotic resistance. In addition, our work unveiled potential pharmacological applications for genotypic antibiotic resistance inherent to the mutations of *glgB* and *oppA* as they epistatically interact with *fprA* and *embC*, two genes recently included as antibiotic-resistant genes in the catalog of the World Health Organization. Our findings showed that this approach allows the identification of relevant epistatic interactions that may lead to a better understanding of *M. tuberculosis* by deciphering the complex interactions of molecules involved in its metabolism, virulence, and pathogenesis and that may be applied to different bacterial populations.

## 1 Introduction

In humans, tuberculosis (TB) is a chronic and highly contagious disease that causes more than 10 million human infections and 1.8 million deaths worldwide every year. The constant arrival of drug-resistant strains complicates its control and eradication (Gupta et al., 2018). This disease is mainly caused by members of the *Mycobacterium tuberculosis* complex (MTBC) (Coscolla and Gagneux, 2014) *via* aerosolized bacteria released by patients with TB (Lerner et al., 2015).

*Mycobacterium tuberculosis* (Mtb) lineages L1–L4 and L7 form a large group of human-adapted strains responsible for the vast majority of global human TB cases, whereas *Mycobacterium africanum* lineages (L5 and L6), which are restricted to humans from West Africa, are phylogenetically linked with the eighth lineage, which comprises various animal-adapted strains (Gonzalo-Asensio et al., 2014).

The first complete genome sequence of Mtb was described in 1998 (Cole et al., 1998). Since then, whole-genome sequencing (WGS) has been applied to a wide range of clinical scenarios, with the potential to revolutionize TB diagnosis, outbreak investigation, development of drugs and vaccines, and to assist in understanding the evolution and pathogenicity of MTBC (Satta et al., 2018). The increase in genomic data in this new era of big data can be considered a great opportunity to continue with the epidemiological surveillance of Mtb associated with the evaluation of genotypic antibiotic resistance. Moreover, it may allow us to unveil new genes with characteristics that lead us to a better understanding of TB.

Recent advances in the scale and diversity of population genomic data for Mtb provide the potential for revealing whole-genome genetic patterns. Statistical methods combined with recent advances in computational structural biology have identified the polymorphic loci (positions inside a genome) under the strongest co-evolutionary pressures or epistatic interactions (Skwark et al., 2017). Such epistatic interactions describe a functional relationship between genes or polymorphic loci (Sackton and Hartl, 2016). Studies of interactions between mutations in Mtb that result in resistance to diverse drugs have suggested that epistasis may be related to multidrug resistance (Trauner et al., 2014; Kavvas et al., 2018). However, the role of epistatic interactions among many regions of the genome under selection in Mtb remains unknown, and further study will contribute to improving our knowledge of TB.

In this study, we analyzed polymorphic loci interactions for epistatic detection in a set of 254 complete reference genomes from Mtb by the use of the model-free method, SpydrPick (Pensar et al., 2019). SpydrPick is based on calculating the mutual information between two polymorphic loci. This well-annotated reference collection integrates genome annotation, gene characterization, and a sequence variation report with a high certainty of genomic location. First, a pan-genome was created using Roary (Page et al., 2015). Then, using AMAS, a pan–genome-wide alignment was obtained by concatenating individual gene alignments. This pan–genome-wide alignment was the input for SpydrPick.

The application of the method to this data set allowed us to reconstruct an epistatic network. The analysis of this network revealed two putative targets of co-selection (*glgB* and *oppA*) associated with Mtb genes related to virulence, pathogenesis, transport system modulators of the immune response, and antibiotic resistance. This work may have relevant applications in the characterization of new genes involved in the worldwide problem of Mtb drug resistance (WHO, 2021).

## 2 Materials and Methods

**FIGURE 1:**
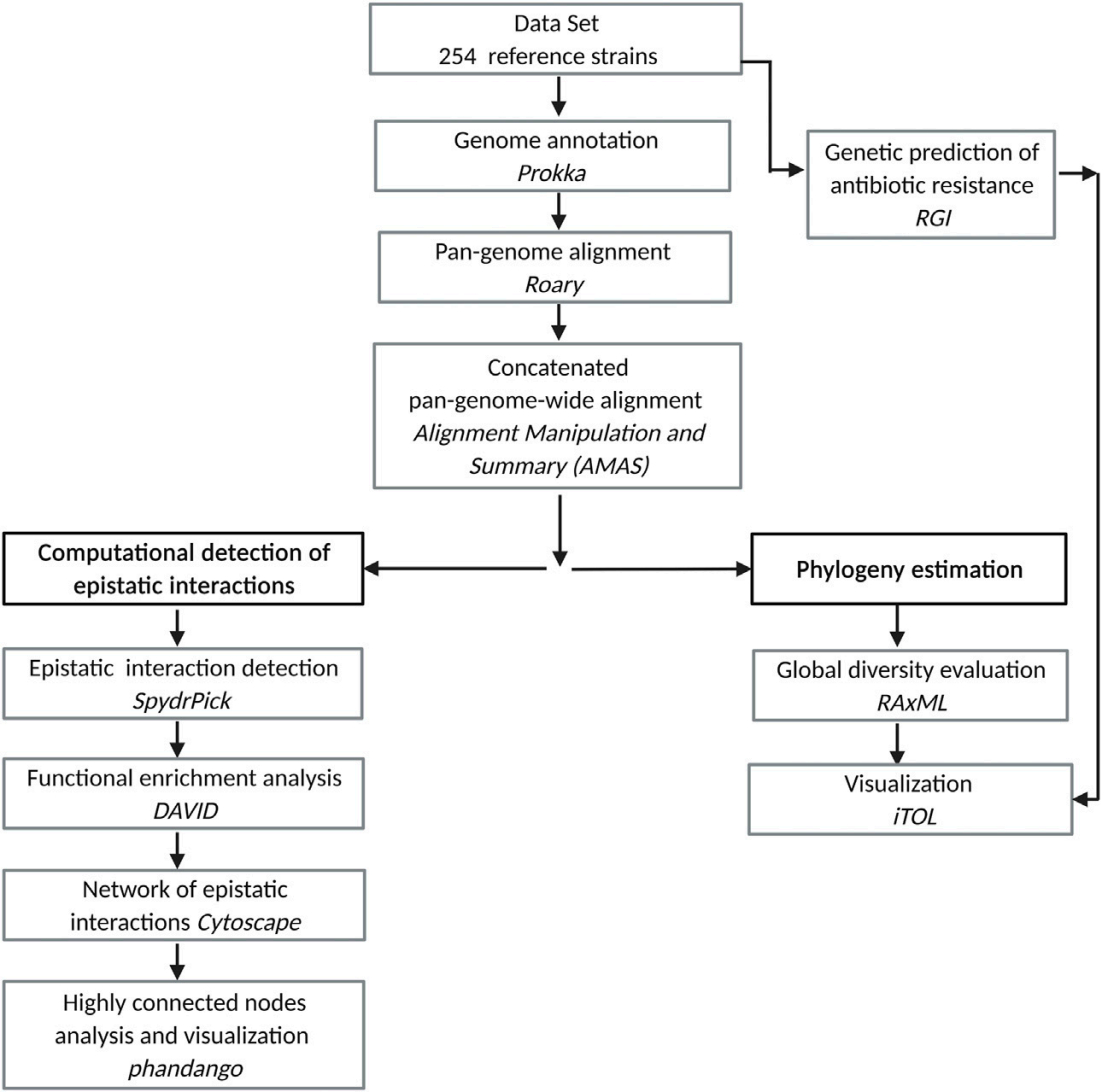
Pipeline for the study of epistatic interactions in Mtb.

An overview of our approach is depicted in Figure 1. The steps are described in the following subsections.

### 2.1 Data Set

We gathered 254 reference strains of Mtb from the NCBI Refseq database that was available as of 4 November 2020. The list of strains is provided as Supplementary Data S1.

### 2.2 Creating Pan–Genome-Wide Alignment

Following the strategy of Pensar et al. (2019), we created a pan–genome-wide alignment of the 254 strains. First, we employed Prokka (Seemann, 2014) to annotate genes and features of interest in the set of strains. This genome annotation (GFF3 format) was the input to create a pan-genome of the strains with Roary (Page et al., 2015).

This tool extracts the gene sequences from the input and then identifies clusters to obtain gene alignments. Roary considers two categories of genes: core and accessory. A gene is considered “core” if it is in at least a certain percentage of strains (isolates) defined by the user. In our study, we followed the approach of Pensar et al. (2019), who set this percentage on 95% strains. The output of Roary is a set of files with individual gene alignments, with one file per gene. These files are concatenated in a matrix using the Alignment Manipulation and Summary (AMAS) tool (Borowiec, 2016). This matrix is formed by gene 1 joined on the right with gene 2 and so on with the rest of the genes [see the example “A: concatenation” from Figure 1 in Borowiec (2016)]. Thus, the columns of the output matrix are the genes, and the number of rows is the number of strains used to generate the pan–genome-wide alignment (254 in this case).

### 2.3 Global Diversity Evaluation

The pan–genome-wide alignment was evaluated for global diversity by estimating a phylogeny using RAxML Next Generation (Kozlov et al., 2019). A standard nonparametric bootstrap of 1,000 replicates was performed. Phylogenies were visualized using iTOL v. 6.4.1 (Letunic and Bork, 2021).

### 2.4 Genetic Prediction of Antibiotic Resistance

We predicted a resistome for the 254 strains using the Resistance Gene Identifier (RGI) tool v. 5.1.1 (Alcock et al., 2020). RGI uses the Comprehensive Antibiotic Resistance Database (CARD) as reference data. Using the output of RGI, we annotated strains for two genotypic characterizations of antibiotic resistance: multidrug-resistant (MDR) strains for those strains with genes resistant to isoniazid and rifampicin and extensively drug-resistant (XDR) strains if they have genes resistant to isoniazid, rifampicin, fluoroquinolone, and at least one of the following three antibiotics: kanamycin, amikacin, or capreomycin. These annotations were incorporated for visualization into the phylogeny displayed by iTOL.

### 2.5 Computational Detection of Epistatic Interactions

#### 2.5.1 Epistatic Interaction Detection

We utilized SpydrPick (Pensar et al., 2019) to detect the epistatic interactions in the pan–genome-wide alignment. SpydrPick is a model-free method whose computational efficiency enables analysis at the scale of pan-genomes of bacteria. This method facilitates the detection of targets of co-selection related to virulence and antibiotic resistance. The potential of this method is the detection of epistatic interactions in the absence of phenotypic data.

The approach of SpydrPick is based on calculating the mutual information (MI) between two polymorphic loci. MI is an information-theoretic measure of the amount of information that one random variable, *X*, contains about another random variable, *Y*. MI is also defined as the reduction in uncertainty in *X* after observing *Y*; in other words, MI manifests the reduction in uncertainty of *X* due to the knowledge of *Y* (Cover and Thomas, 2006). MI gives a measure of association or correlation between *X* and *Y* (Chanda et al., 2020); if the two variables, *X* and *Y*, are independent, then the MI is zero. MI is formally defined as follows:

$$MI\left(X,Y\right)=\underset{x\in \left(X\right)}{\sum }\underset{y\in \left(Y\right)}{\sum }p\left(x,y\right)\log\left(\frac{p\left(x,y\right)}{p\left(x\right)p\left(y\right)}\right),$$ (1)

where *p* (*x*, *y*) is the joint probability and *p*(*x*) and *p*(*y*) are the marginal probabilities of *X* and *Y*. MI has been successfully used for detecting co-selection in bacterial population genomics at a genome-wide scale. Another relevant feature introduced by SpydrPick’s approach is the correction for the population structure. This is applied by a sequence reweighting strategy based on how different are the sequences in the pan–genome-wide alignment (Pensar et al., 2019).

SpydrPick detects direct and indirect interactions between loci. A direct interaction occurs between two positions (*P*_1_ → *P*_2_), whereas an indirect interaction occurs when the two positions (*P*_1_ and *P*_2_) are also linked through a third position (*P*_1_ → *P*_3_ → *P*_2_). In the case of indirect interactions (*P*_1_ → *P*_2_), SpydrPick removes the interaction if the MI is not larger than the other two interactions (*P*_1_ → *P*_3_ and *P*_3_ → *P*_2_).

In addition, SpydrPick performs an analysis to detect outlier interactions. A first criterion to filter outliers is that the distance (bp) between the positions of polymorphic loci must be greater than a linkage disequilibrium (LD) parameter. In this case, a strong LD refers to a close genetic distance between two nucleotide positions. Due to a strong LD hiding a prospective signal of shared co-evolutionary selection pressure, SpydrPick filters out pairs of positions with strong LD to select outlier interactions. According to the SpydrPick’s documentation (https://github.com/santeripuranen/SpydrPick), for bacterial genomes, the typical values of the LD are in the 500–20,000 bp range, and the default approach to filtering out strong LD pairs is using a simple distance-based cut-off (20,000 in our case). The second criterion is that the MI must be greater than a threshold obtained from Tukey’s outlier test *Q*_3_ + 1.5 × (*Q*_3_ − *Q*_1_) (Tukey, 1977).

The output of SpydrPick is a table of epistatic interactions that includes the pair of positions of two interacting polymorphic loci in the pan–genome-wide alignment, the genome distance between the two positions, the type of interaction (direct/indirect), and the MI score. When SpydrPick detects outliers, they are reported in another table, including three additional fields: the MI score without gaps, the gap effect, and if the outlier is considered an extreme outlier (*MI* > *Q*_3_ + 3 × (*Q*_3_—*Q*_1_)). From the input alignment, SpydPick categorizes any character different from A, C, G, and T as a gap. Gaps are considered in the default MI calculation, so *X* and *Y* have an outcome space of five categories. As the gaps may not be informative, SpydrPick calculates for each pair of positions in the outliers another MI score considering only those strains without gaps in either of the two positions. This MI score is named mutual information without gaps (MI_wo_gaps). Using the MI score without gaps, the gap effect is calculated as (1—*MI*_*wo*_*gaps*/*MI*) × 100 to quantify the positive or negative effect on the MI by discarding strains with gaps in the two positions.

Comparing MI scores without gaps in a meaningful way is difficult due to the fact that the set of strains without gaps in the two positions varies between pairs of positions (Pensar et al., 2019). However, a high value of the gap effect for a given pair of positions may indicate a gap-driven interaction, and a manual analysis of the pair should be required. Thus, following the analysis performed by Pensar et al. (2019), we used the default MI, leaving the analysis of the MI_wo_gaps for a future in-depth study.

The loci of epistatic interaction were annotated with gene id and gene name. Gene names were obtained from the partitions generated by AMAS using an R script. Afterward, using another R script (https://github.com/biotb/epitb-net) and the R Biomartr library (Drost and Paszkowski, 2017), we retrieved the ENTREZ gene id by searching the gene name in the GFF file of the Mtb H37Rv reference genome (GCF_000195955.2).

If there is no gene name detected by Roary during the pan-genome creation, then Roary gives a unique generic name formed by the prefix group and a consecutive number. These generic names also appear in partitions of AMAS; however, no ENTREZ id could be associated with these generic names as these names did not exist in the reference genome GFF file.

On the other hand, Prokka was indicated by a numeric suffix different annotation for the same gene, such as carB_1 and carB_2 (carbamoyl-phosphate synthase large chain). These names were also not found in the reference genome GFF file. In these cases, we eliminated the numeric suffix to find the gene name in the reference genome file. For example, we were able to find the gene id 886,253 for *carB*.

#### 2.5.2 Functional Enrichment Analysis

We used the database for annotation, visualization, and integrated discovery (DAVID) v6.8 (Huang et al., 2009) to obtain a functional annotation of Gene Ontology (GO) terms and KEGG pathways of the genes participating in the epistatic interactions. Specifically, we used the DAVID Web Service Python Script (Jiao et al., 2012) to generate a chart report.

#### 2.5.3 Network of Epistatic Interactions

The set of epistatic interactions can be seen as a model of complex epistatic relations that may be analyzed and displayed as a network. Here, we used Cytoscape (Kohl et al., 2011) to study our set of epistatic interactions. This tool has been utilized for studying diverse types of genetic networks. Cytoscape includes an Analyze Network Tool that calculates several network parameters, such as node degree and betweenness centrality. Another useful tool of Cytoscape is the set of layout algorithms based on the yFile Layout Algorithm App. These algorithms visually organized a network by aligning and rotating groups of nodes.

#### 2.5.4 Highly Connected Nodes Analysis and Visualization

We focused on the most highly connected genes (the highest degree) for analyzing our epistatic network. Functional characterization of these genes was performed by literature curation and showing enriched GO terms for genes interacting with them. In addition, we used the R package SeqinR (Gouy et al., 1985) to upload the pan–genome-wide alignment and extract the allele distribution at loci involved in their epistatic interactions. We used the interactive web tool Phandango, which is used to visualize phylogenetic trees and associated genomic information (Hadfield et al., 2017), to show the estimated phylogeny and allele distribution of loci.

## 3 Results and Discussion

### 3.1 Pan–Genome-Wide Alignment

A total of 6,205 individual genes were aligned by Roary, including 3,659 core genes. After concatenating all individual genes with AMAS, a pan–genome-wide alignment of 6,751,593 bp was obtained.

### 3.2 Estimated Phylogeny and Antibiotic Resistance Prediction

Based on Akaike Information Criterion (AIC) and the Bayesian Information Criterion (BIC), which are theoretical information criteria to penalize complex models, we selected the estimated phylogeny using a GTR model with four free rates (GTR-R4-FO). The comparison of models is provided in Supplementary Table S1. Convergence using the extended majority rule (MRE) criterion (Pattengale et al., 2010) with a 3% cutoff for the bootstrapping was reached after 400 trees.

**FIGURE 2:**
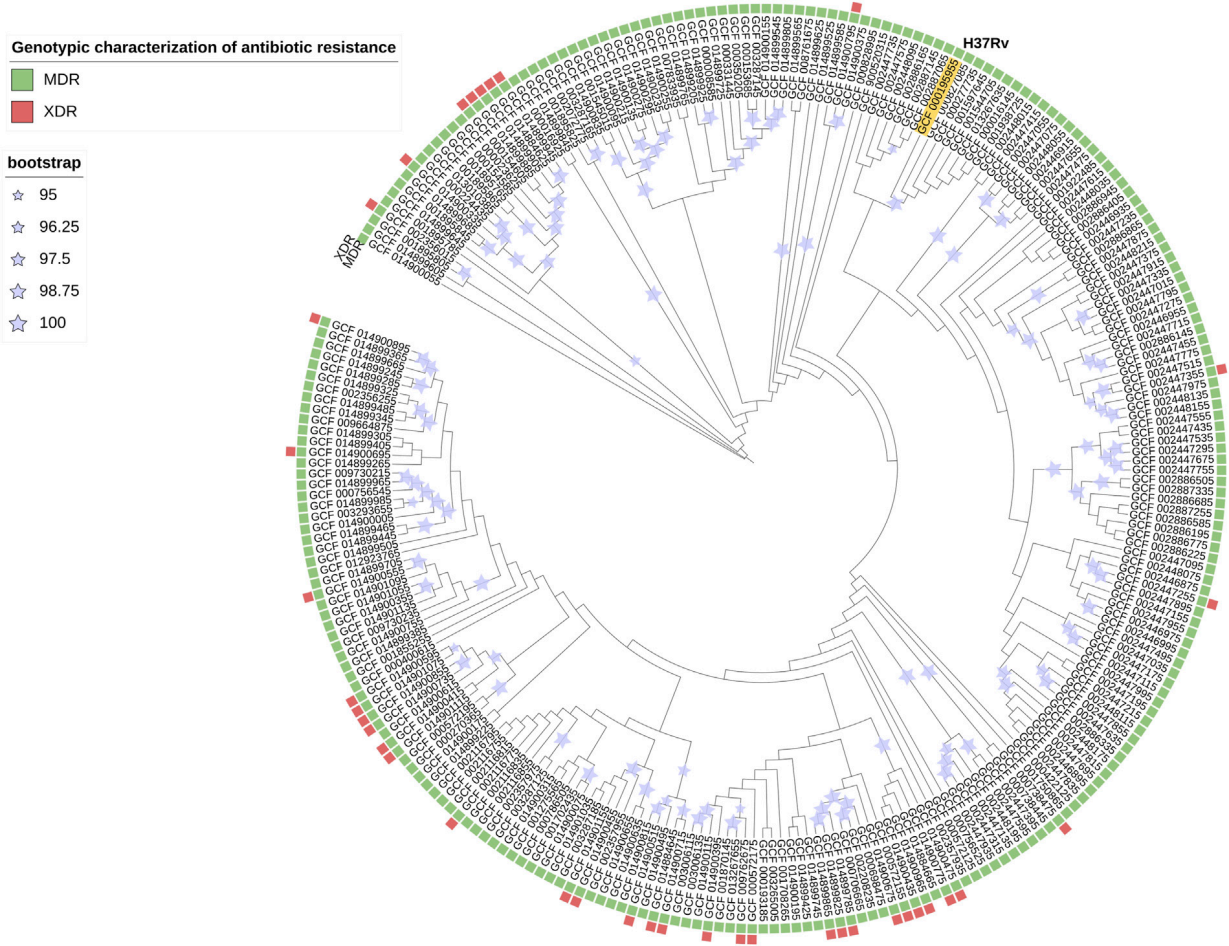
Phylogenetic tree pan-genome Mtb is an iTOL circular visualization with the branch length and the bootstrap values displayed. The tree is based on the Maximum Likelihood topology of 254 strains representative of Mtb diversity and shows that length is proportional to nucleotide topology. Bootstrap values for clades corresponding to the main Mtb clades are shown. The colors correspond to the different genotypic characterizations of antibiotic resistance (MDR = Multidrug Resistant; XDR = Extensively Drug-Resistant).

The prediction of antibiotic resistance by RGI reported that 100% of the 254 strains were MDR (Figure 2) and, within this, 15% were XDR. This result indicates that bacterial strains, perhaps currently circulating, present a high level of resistance to first-line treatments, hindering the successful response to treatment and facilitating the dissemination of strains with drug resistance mutations. Thus, detecting epistatic interactions to elucidate polymorphic loci under the strongest co-evolutionary pressure is of utmost importance for molecular surveillance with bioinformatic tools that help us characterize them promptly. Currently, it is reported that 3.4% of the new TB patients and 20% of the patients with a history of previous treatment for TB were diagnosed with MDR TB worldwide (WHO, 2021).

The phylogenetic tree (Figure 2) shows the nucleotide diversity of Mtb (254 strain collection). In this study, there is heterogeneity of submitters, 100% of the strains present genes linked to antibiotic resistance and with respect to the H37Rv strain (reference), and most of the strains present greater genetic diversity.

### 3.3 Detected Epistatic Interactions

SpydrPick detected 10,573 outlier epistatic interactions (5,484 directed and 5,089 indirect). These interactions describe polymorphic loci under the strongest co-evolutionary pressure. A table with the complete list of outliers is provided as Supplementary Data S2. This table includes the fields described in subsection 2.5.1, that is, the pair of positions of the two interacting polymorphic loci in the pan–genome-wide alignment, genome distance between the two positions, type of interaction (direct/indirect), MI score, MI score without gaps, gap effect, and whether the outlier interaction is considered an extreme outlier.

After gene annotation, we generated a new table of epistatic interactions that excluded the generic gene names given by Roary. The new table of outliers included 1,940 epistatic interactions among 107 unique genes. From this set of genes, we only found 70 in the reference genome GFF file, and they were associated with their ENTREZ id. Filtering only those interactions, including these 70 genes, we obtained a final table with 890 outlier interactions. The remaining interactions that were not considered in our study will be included in a future analysis.

The final table of outlier interactions includes the two positions of the two interacting polymorphic loci in the pan–genome-wide alignment, gene ENTREZ id and gene name for each position, distance between the two positions (bp), type of interaction (direct = 1, indirect = 0), MI score of the interaction, and if the interaction outlier is an extreme outlier (yes = 1, no = 0). This final table of outliers (Supplementary Data S3) was used for enrichment analysis, network reconstruction, and analyses.

SpydrPick was able to find long-distance interactions surpassing the two million bp (Table 1). This fact confirms that our study has a whole-genome scale. However, because we identified epistatic interactions from a pan–genome-wide alignment constructed by concatenating individual gene alignments, the positions are not straightforward whole-genome loci. The minimum distance (bp) between the positions of the two interacting polymorphic loci nearly surpassed the LD criterion of 20,000 bp. The mean of the distance between polymorphic loci in the outlier interactions was 846,454 bp; considering that it is greater than the median (721,980 bp), there may be a slight skewness to distances lower than the mean. On the other hand, the range of MI scores was short, from 0.4130 to 0.5020 (Table 1). The MI scores might show a skewness to low values as the mean (0.4509) was higher than the median (0.4202).

**TABLE 1:** Statistics of the distance between positions of the two interacting polymorphic loci and statistics of the MI scores, both for the final outlier interactions.

Statistic of the final outlier interactions	Values
Minimum distance (bp)	20,870
Maximum distance (bp)	2,328,291
Median distance (bp)	721,980
Mean distance (bp)	846,454
Minimum MI score	0.4130
Maximum MI score	0.5020
Median MI score	0.4202
Mean MI score	0.4509

All loci in the 890 interactions were found in the described single-nucleotide polymorphisms (SNPs) when we used the pan–genome-wide alignment with the tool SNP-sites v. 2.5.1, which can rapidly identify SNPs from a multi-FASTA alignment (Page et al., 2016). This additional step was developed to identify polymorphisms involved in the detected epistatic interactions.

### 3.4 Epistatic Network Analysis

#### 3.4.1 *glgB* and *oppA* as Putative Targets of Co-selection

**FIGURE 3:**
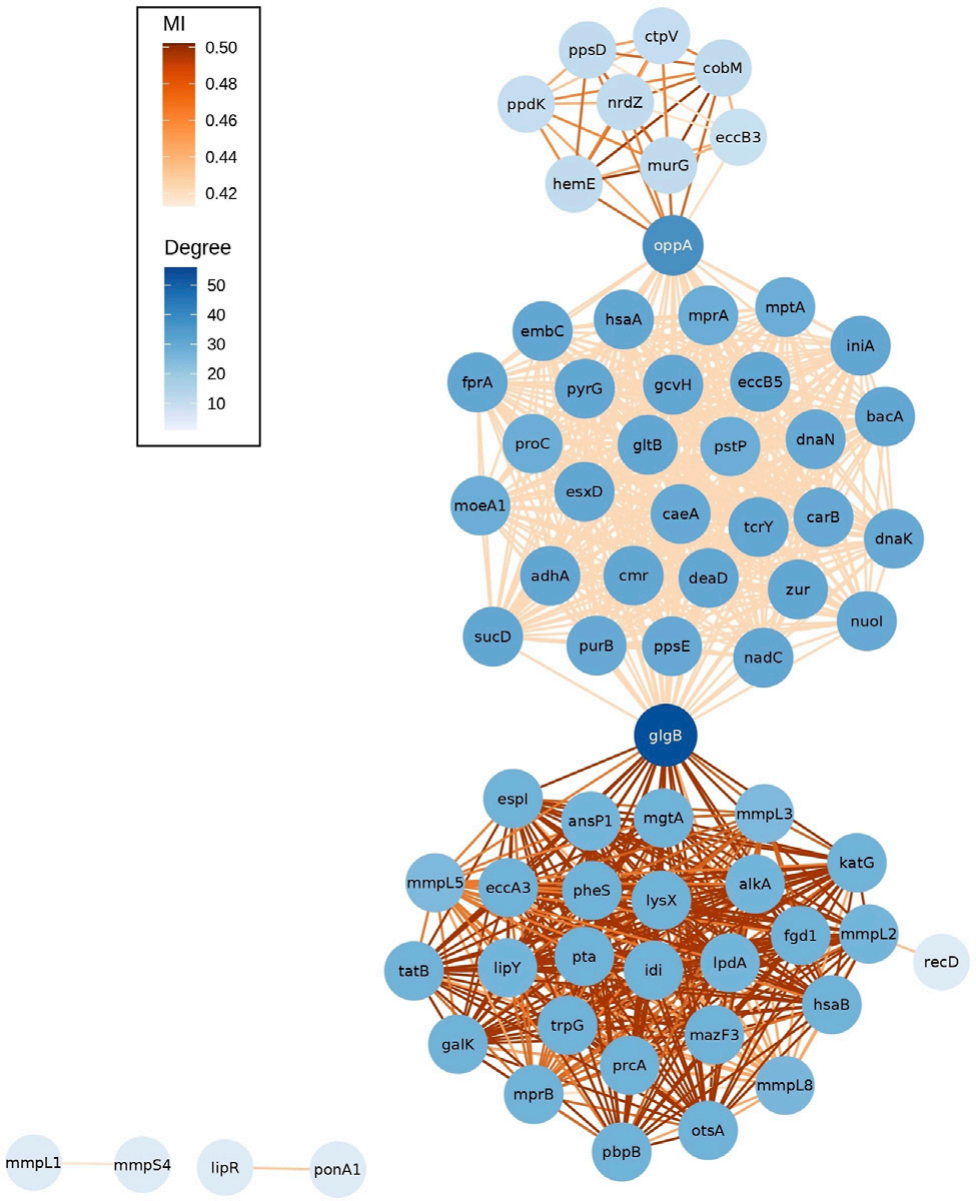
Network of outlier epistatic interactions. The gradient color of nodes depicts the node degree. The gradient color of edges depicts values of MI.

The network of epistatic interactions was analyzed to figure out those genes with a high node degree (the number of edges), that is, a high level of connectivity of the gene with other genes. The most highly connected genes were *glgB* (ENTREZ:886,893, degree = 56), a *α*-1,4-glucan branching enzyme (GlgB), and *oppA* (ENTREZ:886,985, degree = 37), an oligopeptide-binding protein (OppA) (Figure 3).

These two genes also have the highest value of betweenness centrality (*glgB* = 0.493, *oppA* = 0.219). Betweenness centrality is higher for those nodes that join subnets (communities) than those located inside the subnets. Here, we observed three subnets connected by these two genes. An interesting pattern is that each subnet has a different distribution of values of MI (see gradient color of edges in Figure 3). The subnet at the bottom has higher MI values (median MI = 0.502) than the other two, the top subnet has a median MI of 0.463, and the subnet at the middle has lower values (median MI = 0.420). A further study is required to elucidate the cause of this pattern. In addition, future analysis will be required to identify the patterns associated with the isolated subnets (*mmpL1*-*mmpS4* and *lipR*-*ponA1*).

Thus, we consider these two genes as relevant putative targets of co-selection because they may be associated with several genes related to potential pharmacological applications. The GlgB enzyme (encoded by Rv1326c) is the key enzyme involved in the biosynthesis of *α*-glucan, which plays a significant role in the virulence and pathogenesis of Mtb. Recently, enzymes that participate in the biosynthesis of trehalose have gained major attention as drug targets, especially in Mtb (Dkhar et al., 2015), as capsular polysaccharides of bacteria have been found to modulate the host immune response. The importance of the metabolism of GlgB has been described (De Smet et al., 2000), but the epistatic interactions with other genes remain unknown.

On the other hand, the gene *oppA* (oligopeptide-binding protein) works as a substrate-binding protein for the oligopeptide transport system (Opp), which is responsible for peptide importation. The Opp system is an ATP-binding cassette transporter. This helps in peptide absorption, giving pathogens the essential nutrients as a source of carbon, nitrogen, and amino acids. The Opp system affects many cellular processes, including internalization of quorum-sensing peptides, biofilm production, cell surface modification, and antibiotic resistance (Hopfe et al., 2011). The relevance of the characterization of the peptide transporter system has been described by Dasgupta et al. (2010). Previous studies uncovered the novel observation that this peptide transporter modulates the innate immune response of macrophages infected (Cassio Barreto de Oliveira and Balan, 2020) with Mtb, but the epistatic interactions of *oppA* with other loci remain unknown.

A bacterium is able to adapt its response to host conditions, such as intracellular residence in phagocytic cells, oxidative stress, hypoxia, and carbon and nitrogen source. For this reason, evaluating interactions by bioinformatics experiments is necessary for the identification of new epistatic interactions in genes that have been previously reported in databases, such as the catalog of the WHO, or for the understanding of the epistatic interactions in Mtb before the development of new therapies.

#### 3.4.2 Enriched GO Terms for the Epistatic Network

**FIGURE 4:**
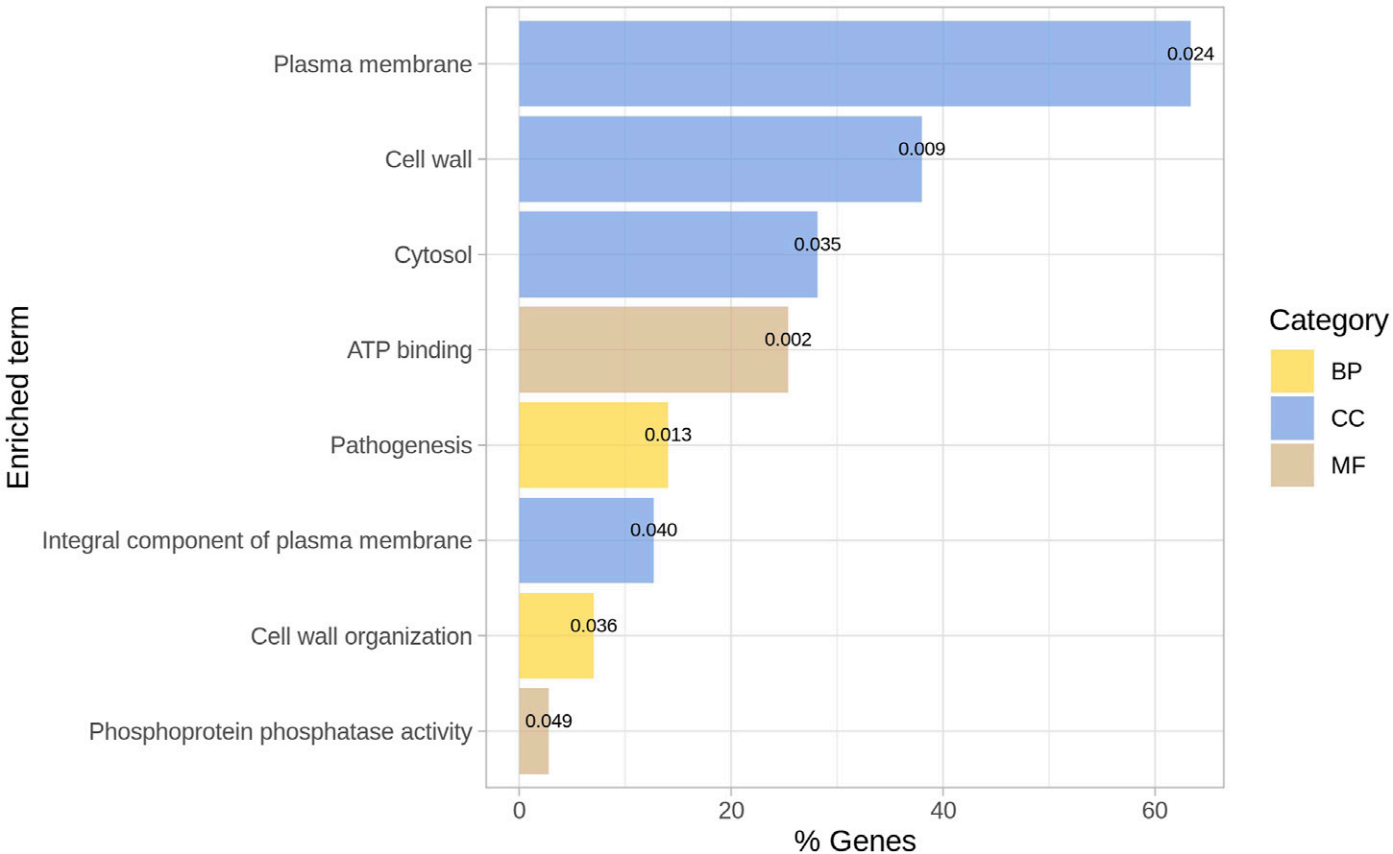
Enriched GO terms for outliers. The *p*-value is indicated for each term. BP = Biological process; CC = Cellular Component; MF = Molecular function.

The list of ENTREZ ids of the genes of the network was used to perform a functional enrichment analysis with DAVID. From the DAVID chart report, we only considered those terms as relevant with *p*-value < 0.05 (see Supplementary Data S4 for details of the functional enrichment analysis). Biological processes of pathogenesis (GO:0009405) and cell wall organization (GO:0071555) were enriched in a subset of genes (Figure 4). The cell wall (GO:0005618), plasma membrane (GO:0005886), cytosol (GO:0005829), and integral components of the plasma membrane (GO:0005887) were the more abundant cellular components; in this case, 63% of the genes are in the plasma membrane. Regarding molecular functions, we obtained enrichment for ATP binding (GO:0005524) and phosphoprotein phosphatase activity (GO:0004721) for some genes.

Recently, the biomarkers of Mtb that regulate immune response have been identified to potentially develop drugs for TB. It has been previously described that the functionality of cellular components was associated with infection and verified the regulation of these cellular components as relevant regulators of the immune response in the host (Li et al., 2020). Thus, describing the genes involved in cellular components is crucial for understanding the interactions of bacteria with host molecules that regulate immune response.

In recent studies, the relevance of the structure and biogenesis-related genes of Mtb encoding glycoconjugates has been confirmed, with particular emphasis on the molecules across the different layers of the cell envelope (Angala et al., 2014). In addition, it has been previously stressed that ATP production is crucial for antibiotic resistance in bacteria (Black et al., 2014).

**FIGURE 5:**
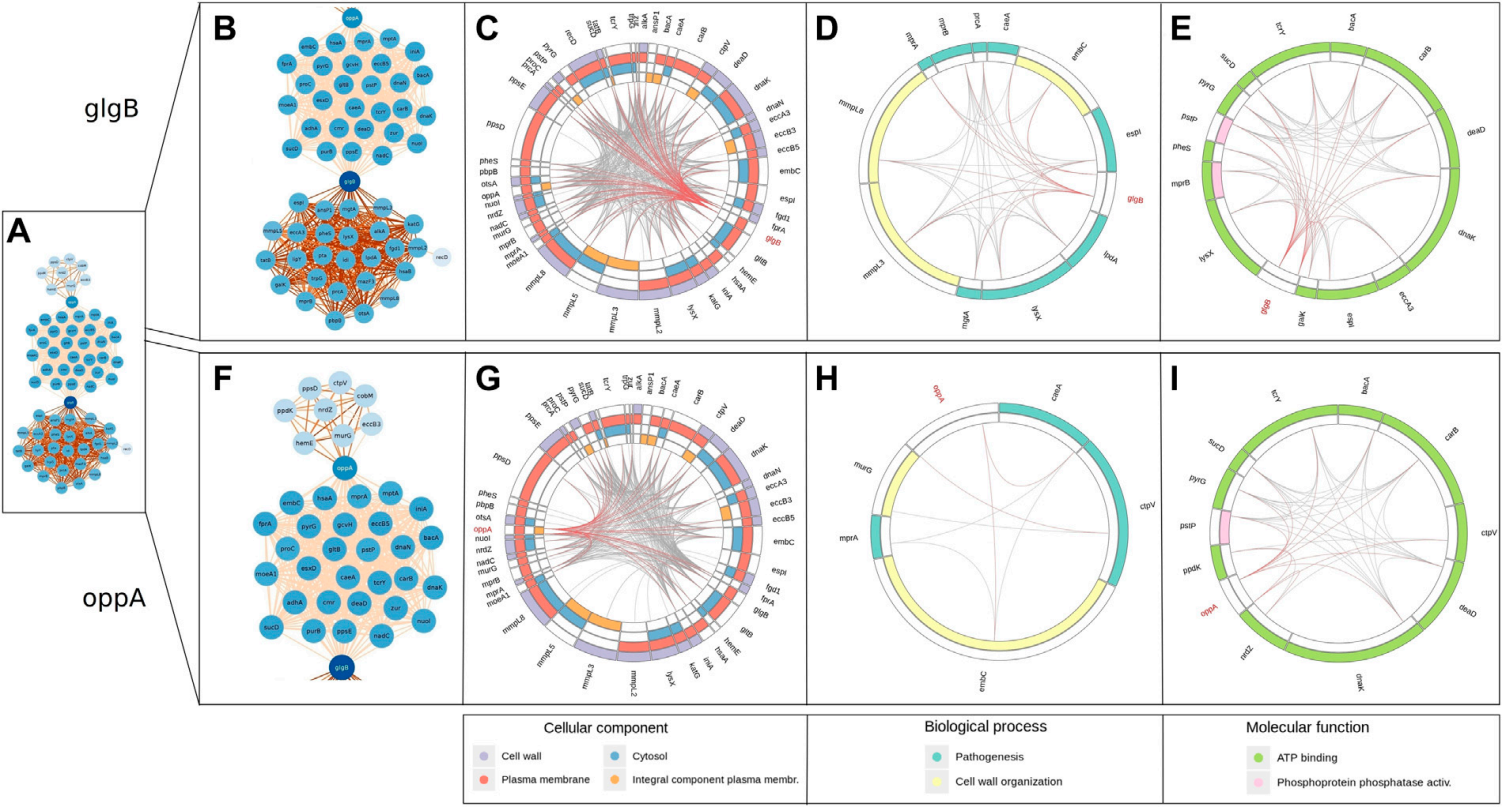
Enriched GO terms of genes that interact with *glgB* and *oppA*. The arrangement and size of genes and positions of interactions reflect the pan–genome-wide alignment. Red lines indicate interactions of *glgB* and *oppA*, whereas gray indicates the interaction of other genes. **(A)** Epistatic network. **(B)** *glgB* interactions (subnet). **(C)** Enriched cellular components of genes interacting with *glgB*. **(D)** Enriched biological processes of genes interacting with *glgB*. **(E)** Enriched molecular functions of genes interacting with *glgB*. **(F)** *oppA* interactions (subnet). **(G)** Enriched cellular components of genes interacting with *oppA*. **(H)** Enriched biological processes of genes interacting with *oppA*. **(I)** Enriched molecular functions of genes interacting with *oppA*.

We show the enriched GO terms of genes interacting with *glgB* (Figure 5B) and *oppA* (Figure 5F) using circular layouts. In addition, Supplementary Data S5 also contains in table format the genes interacting with *glgB*, their product, and enriched GO terms; the same information is provided for *oppA* in Supplementary Data S6. The layouts were generated using the start and end positions of genes reported in partitions generated by AMAS, so the arrangement and size of genes in the layout and positions of interactions reflect the pan–genome-wide alignment. We have highlighted the interactions of *glgB* and *oppA* in red to distinguish them from the interaction of other genes (shown in gray).

Both *glgB* and *oppA* epistatically interact with genes enriched with the biological processes of pathogenesis and cell wall organization (Figures 5D,H). One of these genes is *embC*, which codifies for an arabinosyltransferase involved in the biosynthesis of a major component of the mycobacterial cell wall lipoarabinomannan (LAM). The characteristic manosse-capped LAM of Mtb acts as a pathogen-associated molecular pattern (PAMP), modulating the activation of phagocytic cells to control the strength of the host inflammatory immune response, while representing one of the main components in the cell wall organization. In addition, it has been described that *embC* is expressed as part of a polycistronic mRNA controlled by a promoting region differentially expressed depending on the stationary or hypoxia-induced persistence phase of the bacilli, highlighting the important role of this protein in the biological functions of Mtb and the complex interaction involved in cell wall regulation. Whether or not a direct interaction of *embC* with *glgB* and/or *oppA* exists remains an exciting question to be addressed (Goude et al., 2008).

About molecular functions, *oppA* and *glgB* interact with several genes enriched with ATP binding and with three genes enriched with phosphoprotein phosphatase activity (Figures 5E, I). From these genes, *bacA* is another gene found to be interacting at the highest scores with both *glgB* and *oppA* in the network, and *bacA* encodes for a protein of the type IV family of ABC transporter–type exporters; despite the structure, their function as an importer of multi-solute hydrophilic compounds, such as vitamin B12, bleomycin, and aminoglycosides, has been demonstrated due to a large occluded water-filled cavity that spans across the whole lipid membrane. In addition, it has also been demonstrated that this transporter is implicated in the maintenance of chronic infection in murine models by mediating the transport of a molecule that can directly or indirectly modulate the proinflammatory host response. Despite having different structures, BacA and OppA shared their ability to transport a wide range of substrates; in particular, the shared capacity of import peptides related to the innate immune response suggests a complex regulation and interaction of these transporters, guaranteeing the need to carry out studies at the level of gene regulation and function in the near future (Domenech et al., 2009; Cassio Barreto de Oliveira and Balan, 2020; Rempel et al., 2020).

The enriched GO terms that may be related to those associated with ATP synthase in mycobacteria are of particular interest because they contribute to efficient ATP production, and this enzyme has been validated as a target for potential pharmacological applications. In addition, mycobacterial ATP synthase and its characteristics may provide information on adaptations of bacterial energy metabolism. Mtb can survive in human macrophages for an extended time. For Mtb and other pathogenic mycobacteria strains, the blocking of ATP hydrolysis is relevant as it may represent an adaptation to its internal and external human phagosomes, where ATP, once produced, must not be used (Lu et al., 2014). Thus, the importance of epistatic interactions associated with ATP production in pathogenic bacteria may face exceptional challenges as a variety of pathogens need to deal with low energy conditions, such as low oxygen tensions or nutrient limitation inside the host.

Regarding gene interactions enriched with phosphoprotein phosphatase activity, both *oppA* and *glgB* established epistatic interaction with the *pstP* gene, which encodes the Serine/Threonine Protein Phosphatase PstP of Mtb. Signal sensing and transduction *via* phosphorylation and dephosphorylation of specific target proteins are essential for the survival of both eukaryotic and prokaryotic organisms. In the case of Mtb, 11 serine/threonine protein kinases have been described, but only the serine/threonine phosphatase, PstP, has been identified, highlighting the central role of this protein in the control of vital processes as a negative regulator of kinase activity and global serine and threonine phosphorylation (Iswahyudi et al., 2019).

Recently, other functions of PstP as a regulator of cell wall synthesis and cell division by dephosphorylation of key substrates implicated in both pathways have been described (Sharma et al., 2016). PstP is co-transcribed in an operon with genes involved in peptidoglycan synthesis, with protein kinases PknA and PknB that regulate cell growth and cell division and with *fhaA* and *fhaB*, which encode phosphothreonine recognition proteins that also regulate cell growth and cell division. The involvement of *pstP* with elements necessary for cell wall biosynthesis and their strict dependence on Mn^2+^ for function suggests that the interaction encountered by the computational approach could serve as a starting point for initiating investigations into the molecular interactions that regulate these common processes.

PstP is present as a transmembrane phosphatase and contains a 240–amino acid intracellular catalytic domain, tethered *via* a single transmembrane helix to the 196-amino acid-long extracellular domain (Boitel et al., 2003); it remains to be elucidated if during infection and activation of the innate immune responses (e.g., respiratory burst activation), the degraded bacteria retain the phosphatase activity in the membrane fragments, and these could contribute to the dephosphorylation of the signaling pathways of the innate system, contributing to the evasion of the immune response.

Four cellular components were enriched for genes interacting with *glgB* (Figure 5C) and *oppA* (Figure 5G). The majority of genes encode proteins in the plasma membrane. For example, four genes of the group of mycobacterial membrane protein large (MmpL), *mmpL2*, *mmpL3*, *mmpL5,* and *mmpL8* have epistatic interactions with *glgB* and *oppA*. MmpL proteins export cell envelop components (such as virulence-associated lipids and siderophores) to the periplasmic space, contributing at a high level to the persistence of Mtb in the host (Melly and Purdy, 2019). A further study will be required to investigate the fine regulation between the import and export systems of genes identified under epistatic interaction by our approach in order to establish their relevance and biological implications.

#### 3.4.3 Allele Distribution at Loci of Genes Interacting With *glgB* and *oppA*

To observe the patterns of alleles of the interacting polymorphic loci, we show the allele distribution at interacting loci with the loci of *glgB* and *oppA* using Phandango (Hadfield et al., 2017). SpydrPick detected that three loci of *glgB* (837,764, 839,047, and 839,053) interact with 57 polymorphic loci of 56 genes. For *oppA*, two polymorphic loci (5,934,914 and 5,936,231) were found interacting with 38 loci of 37 genes. Tables with loci and genes are available in the Supplementary Datas S7, S8.

**FIGURE 6:**
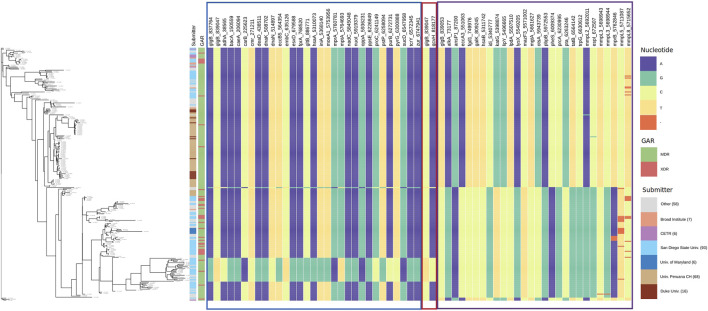
Allele distribution at the loci of genes interacting with *glgB*. Labels of the column indicate gene name and locus. Estimated phylogeny is included on the left. Interacting loci are organized in sections with borders. The border color corresponds to the color of the sections in Supplementary Data S7. GAR = Genotypic Antibiotic Resistance.

**FIGURE 7:**
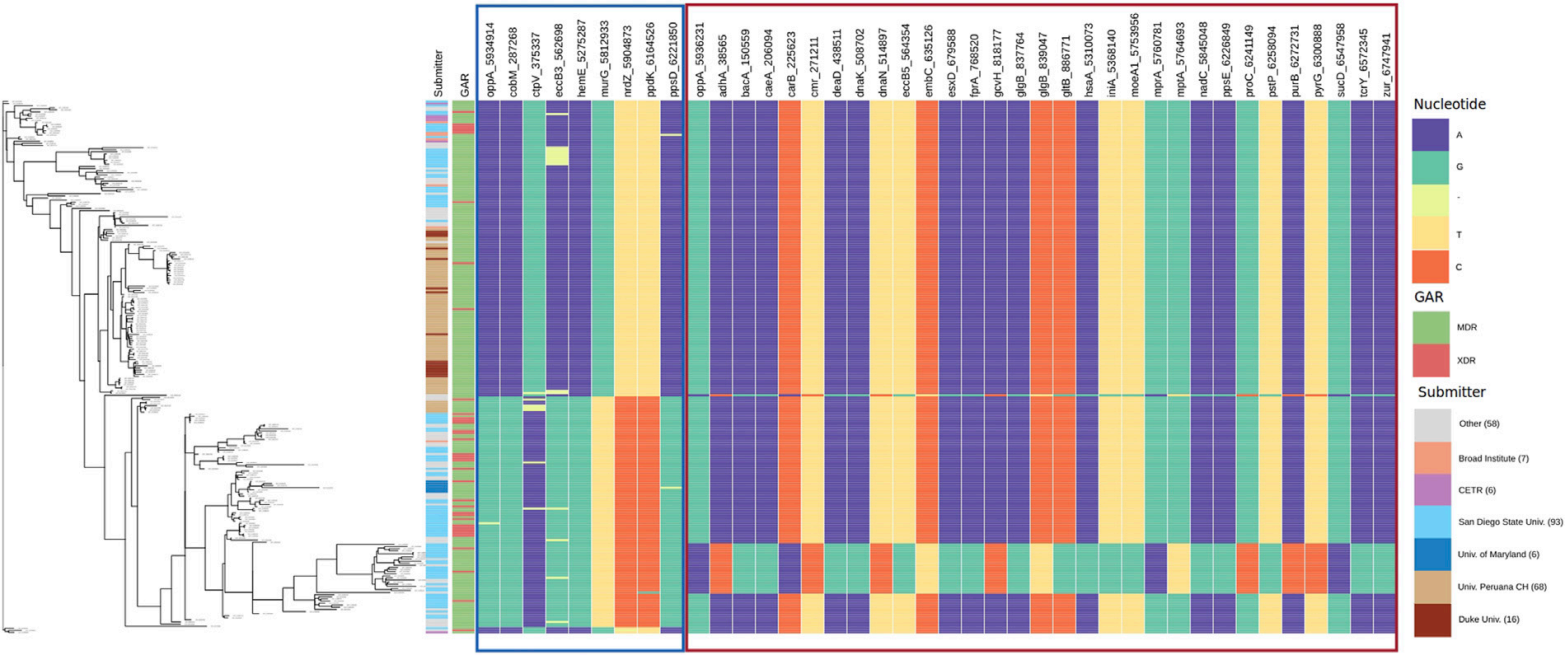
Allele distribution at the loci of genes interacting with *oppA*. Labels of the column indicate gene name and locus. Estimated phylogeny is included on the left. Interacting loci are organized in sections with borders. The border color corresponds to the color of the sections in Supplementary Data S8. GAR = Genotypic Antibiotic Resistance.

Interacting loci and gene names are displayed as labels of columns in Figure 6 for *glgB* and in Figure 7 for *oppA*. Interacting loci are organized in sections with borders. Each section includes the interactions for each interacting locus. The border color for each section corresponds to the color of the sections in Supplementary Data S7, S8. In Figure 6, the first section starts with the loci 837,764 and 839,047 of *glgB* (glgB_837764 and glgB_839047) followed by the 29 polymorphic loci that interact with them, that is, these two loci epistatically interact with each one of the 29 loci.

By observing the allele distribution of pairs of loci, we confirm that SpydrPick is able to detect, using the MI score, predictable patterns of alleles at the two loci. See, for example, the second section in Figure 6, which only depicts the allele distribution of the locus 839,047 of *glgB* (glgB_839047) and the interacting locus 818,177 of *gcvH* (gcvH_818177). It can be noticed that when there is a C in the locus glgB_839047, there is an A in the locus gcvH_818,177, and when there is a T in the locus glgB_839047, there is a C in the locus gcvH_818,177. This predictability is quantitatively depicted by the value of *MI* = 0.4201. The third section in Figure 6 exposes the allele distribution of interactions between the locus 839,053 of *glgB* (glgB_839053) and 27 loci of 26 genes (two loci of the gene *mmpL3* interact with the locus glgB_839053). In this section, we observe gaps (-) in the allele distribution of the interacting locus 5,762,846 of *mprB* (two component histidine-protein kinase/phosphatase MprB, *MI* = 0.4743), locus 5,711,087 of *mmpL5* (transmembrane transport protein MmpL5 *MI* = 0.4670) and locus 5,715,652 of *mmpL8* (integral membrane transport protein MmpL8 *MI* = 0.4435).

In Figure 7, we present the allele distribution of loci interacting with loci 5,934,914 and 5,936,231 of the gene *oppA*. The first section contains the interactions with the locus oppA_5,934,914. We notice the presence of gaps in the positions ctpV_375337 (*MI* = 0.4516) and eccB3_562,698 (*MI* = 0.417). Figure 7 also presents well-defined patterns of allele distribution between interacting loci.

The first column in both figures indicates the submitter institution (we included institutions with less than six submitted strains in the category other), and the second column points to genotypic antibiotic resistance (GAR) predicted with RGI. Estimated phylogeny is included on the left to show the diversity of the strain collection that we analyzed. For example, a clade at the bottom of the tree stands out due to its change of nucleotide in relation to the rest of the strains.

The application of this methodology also allowed the identification within the network of various loci in genes associated with resistance. Recently, the WHO published the first catalog of resistance-associated genetic variants for predicting relevant resistance phenotypes based on more than 38,000 WGS phenotyped isolates (WHO, 2021). This has allowed the identification of multiple positions associated with resistance and their classification into five groups. We use this recent classification to find antibiotic-resistant genes in the epistatic network (Table 2).

**TABLE 2:** Antibiotic-resistant genes reported by the WHO catalogue are found in the epistatic network.

Gene	Antibiotic-resistant gene (drug)
*glgB* and *oppA*	*fprA* (AMI)
*glgB* and *oppA*	*fprA* (CAP)
*glgB* and *oppA*	*embC* (EMB)
*glgB*	*katG* (INH)
*glgB*	*fgd1* (DLM)
*glgB*	*mmpL5* (BDQ)
*glgB*	*mmpL5* (CFZ)

a AMI = amikacin; BDQ = bedaquiline; CAP = capreomycin; CFZ = clofazimine; DLM = delamanid; EMB = ethambutol; INH = isoniazid WHO (2021).

In our network, an interaction was found between *glgB* and *katG*; mutations conferring monoresistance to isoniazid (INH) are common due to INH having been in clinical use since the 1950s. Nevertheless, INH resistance testing is only recently included in some specialized cartridges (e.g., Xpert MTB/XDR) and is not routinely available in such a way that if INH resistance is not detected, patients are treated as pan-suceptible, which represents a high risk of treatment failure and a greater propensity to acquire further resistance (Sulis and Pai, 2020).

**FIGURE 8:**
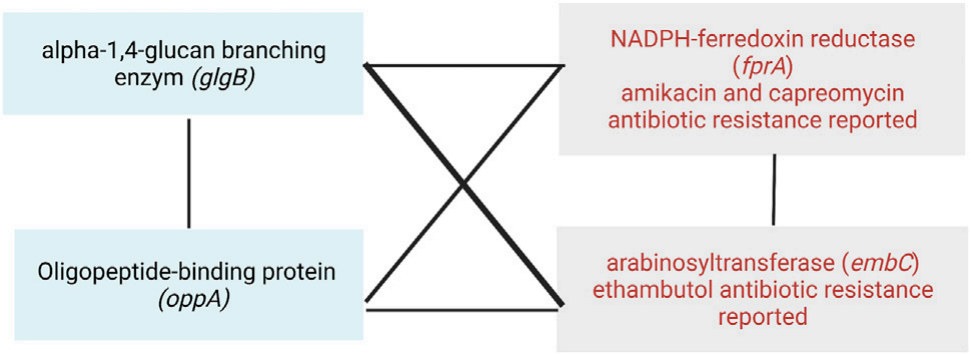
Epistatic interactions between the antibiotic-resistant genes *fprA* and *embC*, and the putative targets of co-selection genes *glgB* and *oppA*.

From the antibiotic-resistance genes that we found in the WHO catalog, we observed that *fprA* (resistant to amikacin and capreomycin) and *embC* (resistant to ethambutol) interact with both genes *glgB* and *oppA* (Table 2). Moreover, they interact between them, forming a clique of four genes (Figure 8). A clique depicts a network where all nodes are fully connected to each other, creating a strong interaction mechanism. This kind of epistatic interactions motivated us to visualize future studies to test new experimental hypotheses to elucidate their biological and pharmacological explanations, and the MI score seems to be a very successful approach to drive so.

## 4 Conclusion

Here, we have presented the reconstruction and analysis of an epistatic network for Mtb from a pan–genome-wide alignment by using the model-free method SpydrPick. Our approach allowed us identifying new epistatic interactions with implications in virulence, pathogenesis, transport system modulators of the immune response, and genotypic antibiotic resistance. By the analysis of the epistatic network, we identified *glgB* and *oppA* as putative targets of co-selection. These two genes epistatically interact with *fprA* and *embC*, two antibiotic-resistant genes reported in the catalog of the WHO, as resistant to ethambutol (*embC*) and amikacin and capreomycin (*fprA*). Our results highlight the importance of implementing computational approaches to elucidate new genes associated to putative epistatic interactions in Mtb.

## Data Availability

The original contributions presented in the study are included in the article/Supplementary Material; further inquiries can be directed to the corresponding authors. R scripts are available at https://github.com/biotb/epitb-net.
